# An Unusual Presentation of Thyrotoxicosis: Leg Weakness and Hypokalemia in a 21-Year-Old Male

**DOI:** 10.1155/2021/1776538

**Published:** 2021-10-23

**Authors:** Mario S. Campana, Maria Riofrio, Raja S. Jadav, Mattan Rozenek

**Affiliations:** St. Barnabas Hospital, Department of Internal Medicine, SBH Health System, The Bronx, NY 10457, USA

## Abstract

Patients with hyperthyroidism or thyrotoxicosis present with an unspecific constellation of signs or symptoms such as palpitations, tremors, weight loss, or diarrhea. In some severe cases, hyperthyroidism can predispose patients to metabolic abnormalities and arrhythmias. Thyrotoxic periodic paralysis (TTP) is a rare, life-threatening complication or variant of hyperthyroidism associated with hypokalemia and muscle weakness that affects young Asian or Hispanic males between 20–40 years. TTP is reversible, and the management consists of beta-blockers, antithyroid therapy, and conservative potassium correction to prevent severe cardiovascular events such as ventricular arrhythmias with the improvement of transient muscle paralysis. We present a case of a 21-year-old Hispanic male complaining with symptoms of thyrotoxicosis, marked hypokalemia, and severe generalized muscle weakness. Physicians must be aware of this uncommon complication of thyrotoxicosis called thyrotoxic periodic paralysis (TTP) to avoid potassium overcorrection and all the endocrine associations with this pathology.

## 1. Introduction

Thyrotoxic periodic paralysis is a rare manifestation or complication of thyrotoxicosis that usually affects a young group of patients between 20 and 40 years [1, 2]. The incidence is approximately 2% in the Asian population and about 0.1–0.2% in North American. It appears to be more common in men than in women, with almost a 26 : 1 difference, which is unusual because thyrotoxicosis has a higher incidence among women. Association between testosterone levels and increased Na/K+ ATPase pump action explain this predilection to males [3].

TTP is described as an onset of sporadic episodes of weakness and muscle pains seen predominantly at resting periods, usually at night, that worsens with physical activity, high-carbohydrate meals, exercise, and stress. It mainly affects the lower extremities and, less commonly, the upper extremities depending on the severity [4].

Hypokalemia in TTP is secondary to an intracellular shift of potassium induced by the increased levels of thyroid hormones that sensitize the Na+/K+ ATPase pumps. Obesity, hyperinsulinemia, or excessive levels of testosterone have been associated as triggers of TTP in patients with hyperthyroidism, increasing the activity of Na+/K+ pump and giving more predisposition to males [3, 5, 6].

The diagnosis of TTP can be challenging and misdiagnosed with other genetic syndromes such as Familial Hypokalemic Periodic Paralysis (FHPP) because of their similar clinical presentation and electrolytes imbalance.

Unlike TTP, familial hypokalemic periodic paralysis is an autosomal dominant condition that develops in patients with positive family history without features of hyperthyroidism.

The treatment of TPP includes acute management of thyrotoxicosis with beta-blockers, antithyroid therapy, and cautious potassium correction. Once the patient is euthyroid and normokalaemia was reached, muscular symptoms will subside. On the other hand, FHPP treatment is based on potassium replacement [7–9]. An accurate diagnosis is important to prevent life-threatening complications such as ventricular arrhythmias, constant muscular paralysis, and potassium overcorrection.

## 2. Case Presentation

A 21-year-old Hispanic male patient without any past medical history presented to the emergency department with a persistent, gradual, and generalized muscle weakness and sprain of the lower and upper extremities that started one week ago but was progressing from 5 to 10/10 of intensity and worsening with physical activity. The patient reported proximal muscle weakness with difficulty walking, standing up, and lifting objects. He had similar symptoms in the past but never this severe. He reported mild palpitations without chest pain, shortness of breath, blurry vision, weight loss, nausea, vomiting, or diarrhea.

The patient denied any family history of electrolytes imbalance, diabetes, hypertension, or thyroid disease and denied allergies or surgeries in the past but used alcohol and marijuana occasionally.

**Figure 1:**
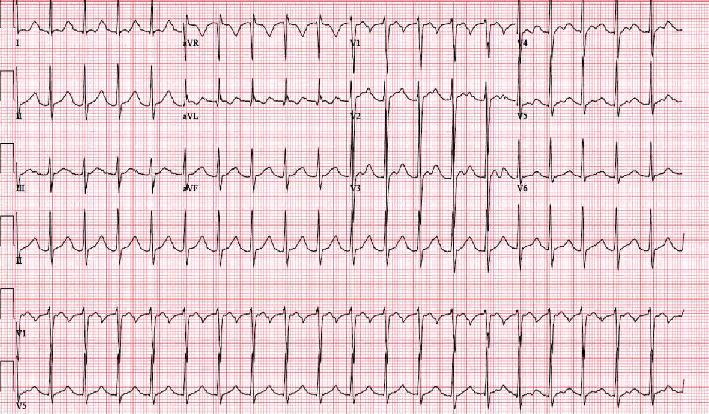
EKG on admission.

On examination, he was anxious, agitated, and with moist skin; vital signs showed a blood pressure of 133/85 mm Hg, heart rate 115 beats/minute, RR 22 breaths/minute, and temperature of 98.0 Fahrenheit. Head exam did not show lids lag, retractions, exophthalmos, or thyromegaly. Cardiopulmonary exam showed regular heart sounds with tachycardia but negative for crackles or wheezing and good work of breathing. Neurologic exam revealed normal sensation and deep tendon reflex but mild fine tremors on his hands with decreased strength in both lower extremities 2/5 and 3/5 in the upper extremities with preserved respiratory muscles. Cranial nerves 2–12 preserved. Electrocardiogram (EKG) showed sinus tachycardia without ST changes or T-wave inversions but prolonged QT interval (Figure 1). Laboratory findings including troponin, glucose, bicarbonate, renal profile, sodium, and calcium were within normal limits. Serum potassium was 2.1 mEq/L, magnesium 1.4 mEq/L, VBG was with an LA of 2.9 mmol/L, and CPK 1569 IU/L. The thyroid function test showed T4 19.4 ug/L (normal: 5–12 ug/L) and TSH 0.01 IU/mL (normal: 0.35–5.60 IU/mL) with normal cortisol levels (Table 1). Urine potassium was 66 (2–300 mEq/L).

The patient was managed with intravenous fluids, propranolol (60 mg daily), PTU (100 mg every 8 hour), and potassium chloride of 120 mEq oral and 40 mEq intravenous doses on the day of admission to the intensive care unit.

**Figure 2:**
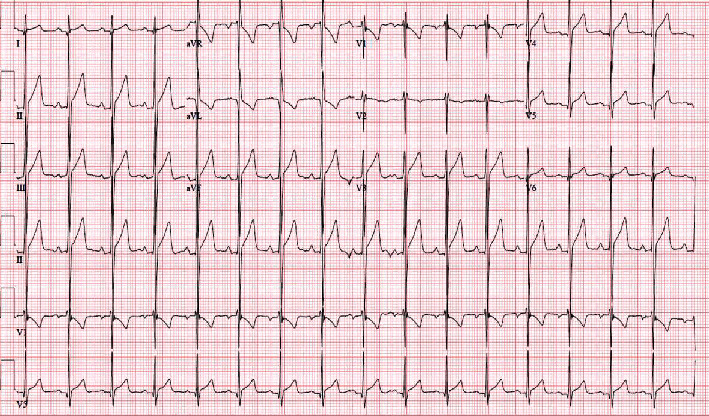
EKG on the second day of admission.

On the second day of hospitalization, potassium levels had normalized, and the patient's weakness subsided. Subsequent EKG (Figure 2) showed resolution of the QT prolongation and tachycardia. The motor strength at this time was 5/5 in the upper and lower extremities. The endocrinology team was consulted and recommended continuing beta-blockers (60 mg daily) and started on methimazole (10 mg daily) with conservative potassium correction and monitoring every 12 hours.

On the last day of hospitalization, his weakness has totally resolved, and the potassium levels had normalized. The patient requested his discharge and stated he will follow-up in the endocrinology clinic for further hyperthyroidism work up as an outpatient. He was discharged with the same dose of methimazole and propranolol.

## 3. Discussion

Hyperthyroidism is associated with some muscular complications, including thyrotoxic myopathies, myasthenia gravis, exophthalmic ophthalmoplegia, and TTP. TTP is characterized by a triad of muscle weakness, hypokalemia, and hyperthyroidism. The etiology of TTP is related to the increased activity of NA+/K+ ATPase pumps on the skeletal muscle [3, 10]. TTP has more predispositions to men compared to women despite thyroid disease being more associated with female patients. This strong predilection that occurs in males showed a possible association between increased testosterone levels and hypokalemic paralysis in patients with hyperthyroidism [3]. Androgens can increase the expression and activity of the Na+/K+ ATPase, therefore correlating with the severity of hypokalemia [11, 12].

Patients with a history of hyperthyroidism, obesity, type 2 diabetes mellitus, or metabolic syndrome with an increased risk of insulin resistance and subsequent hyperinsulinemia are more likely to develop hypokalemic muscle paralysis in acute thyrotoxicosis, creating a potential relationship between insulin resistance and TTP. In the study by Soonthorpun et al. [13], they found that subjects with a history of TTP were more obese and had lower insulin sensitivity than those with a history of simple thyrotoxicosis; therefore, insulin resistance with compensatory hyperinsulinemia may be a key feature of the pathogenesis of TTP [4, 13, 14]. There is no clear risk factor or etiology for TTP, but hyperinsulinemia or conditions that increase the levels of testosterone and catecholamine can increase the likelihood of developing TTP in a patient with thyrotoxicosis. The clinical characteristic of TTP is recurrent transient episodes of proximal muscle weakness, usually more in the lower extremities with spared sensory function. Paralysis can last from a few hours up to 48–72 h, with almost complete recovery after beta-blocker and potassium therapy. Some of the features of this condition can mimic acute neurologic syndromes such as Guillen Barre, transverse myelitis, or spinal cord lesions, but TTP can lead to spared respiratory muscles paralysis. Most patients have decreased or absent deep tendon reflex, which is discordant to other forms of hyperthyroidism [3, 10]. TTP is related with a normal acid-base balance, but with some electrolytes abnormalities such as hypophosphatemia, hypomagnesemia, low potassium excretion rate, and low trans-tubular potassium gradient (TTKG) [14].

There are some genetic etiologies that can share the same pattern of muscle weakness and electrolyte abnormalities of TTP. Familial hypokalemic periodic paralysis (FHPP) is a rare autosomal dominant disease caused by a mutation in either the L-type calcium channel or voltage-gated sodium channel. FHPP, as well as TTP, can present with episodes of low serum potassium and proximal muscle weakness. This disorder has lower clinical expression in females compared to males, with a male predominance of 9 to 1 [3, 7, 14]. Except for the hyperthyroidism features on TTP and the strong family story in FHPP, both entities are difficult to differentiate because of their similar clinical presentation of transient muscle weakness and severe hypokalemia with potential cardiogenic complication. However, both conditions have a different therapy approach. FHPP treatment consists of alleviating the symptoms, preventing acute attacks, and appropriate correction of potassium [14, 15].

On the other hand, the management of TTP consists of keeping the patient on a euthyroid state, with beta-blockers and thioamides, monitoring and avoiding life-threatening cardiogenic complications such as ventricular arrhythmias with a conservative correction of potassium to avoid paradoxical hypokalemia or rebound hyperkalemia. Propranolol inhibits the activity of Na+/K+ ATPase and, thus, increasing the levels of potassium, sometimes without the need for an aggressive replacement. Propranolol and high dose of antithyroid therapy allow a rapid resolution of symptoms and decrease the risk of rebound hyperkalemia.

## 4. Conclusions

TTP is a rare but potentially life-threatening complication of acute thyrotoxicosis.

It is important to assess patients with potential risk for developing TTP during clinical visits and identifying a history of obesity, metabolic syndrome, type 2 diabetes, or excessive testosterone levels. Propranolol and antithyroid therapy with a conservative and limited replacement of potassium are the key factors for appropriate management of TTP in the ICU.

This case report has a clinically and management relevance in patient with high risk of developing TTP and with atypical presentation of thyrotoxicosis. Endocrinologists, internists, and critical care physicians need to be aware and assess these complications during the admission.

## Figures and Tables

**Table 1:** Pertinent laboratory values on admission.

Labs	Value	Reference
Potassium	2.1 (mEq/L)	(3.5–5.3 mEq/L)
Magnesium	1.4 (mEq/L)	(1.3–2.1 mEq/L)
TSH	0.01 (IU/mL)	(0.34–5.60 IU/mL)
Total T4	19.4 (ug/L)	(6.0–12.0 ug/L)
T3	14.9 (pg/mL)	(2.0–4.4 pg/dL)
FT4	4.59 (ng/dL)	(0.82–1.77 ng/dL)
CPK	1569 IU/L	(38–174 IU/L)

## Data Availability

All the information is present in the manuscript.

## Abbreviations

TTP: Thyrotoxic periodic paralysis
FHPP: Familial hypokalemic periodic paralysis
EKG: Electrocardiogram.
